# Phylogenetic and functional potential links pH and N_2_O emissions in pasture soils

**DOI:** 10.1038/srep35990

**Published:** 2016-10-26

**Authors:** M. d. Sainur Samad, Ambarish Biswas, Lars R. Bakken, Timothy J. Clough, Cecile A. M. de Klein, Karl G. Richards, Gary J. Lanigan, Sergio E. Morales

**Affiliations:** 1 Department of Microbiology and Immunology, Otago School of Medical Sciences, University of Otago, Dunedin, New Zealand; 2 Department of Environmental Sciences, Norwegian University of Life Sciences, Ås, Norway; 3 Department of Soil and Physical Sciences, Lincoln University, Lincoln, New Zealand; 4 AgResearch Invermay, Mosgiel, New Zealand; 5 Teagasc, Environmental Research Centre, Johnstown Castle, Wexford, Ireland

## Abstract

Denitrification is mediated by microbial, and physicochemical, processes leading to nitrogen loss via N_2_O and N_2_ emissions. Soil pH regulates the reduction of N_2_O to N_2_, however, it can also affect microbial community composition and functional potential. Here we simultaneously test the link between pH, community composition, and the N_2_O emission ratio (N_2_O/(NO + N_2_O + N_2_)) in 13 temperate pasture soils. Physicochemical analysis, gas kinetics, 16S rRNA amplicon sequencing, metagenomic and quantitative PCR (of denitrifier genes: *nirS, nirK, nosZI and nosZII*) analysis were carried out to characterize each soil. We found strong evidence linking pH to both N_2_O emission ratio and community changes. Soil pH was negatively associated with N_2_O emission ratio, while being positively associated with both community diversity and total denitrification gene (*nir* & *nos*) abundance. Abundance of *nosZII* was positively linked to pH, and negatively linked to N_2_O emissions. Our results confirm that pH imposes a general selective pressure on the entire community and that this results in changes in emission potential. Our data also support the general model that with increased microbial diversity efficiency increases, demonstrated in this study with lowered N_2_O emission ratio through more efficient conversion of N_2_O to N_2_.

The Anthropocene has resulted in a loss of global biodiversity and enhanced greenhouse gas emissions^1^. A major driver of change has been the transformation of land for agriculture purposes, needed to sustain the expanding global populations^2^. These changes are expected to drive further reductions in biodiversity and the loss of associated ecosystem services^3^. Of the greenhouse gases associated with agriculture, nitrous oxide (N_2_O) is of particular concern due to its global warming potential (>300 times more powerful as CO_2_) and ozone-depleting capabilities^4^,^5^,^6^,^7^.

The mechanisms that control N_2_O production and loss from soils are still being debated, with identified regulators comprising physical, chemical and biological factors^8^. Soil pH has been identified as a master regulator of gaseous N emissions, with the propensity of soils to release N_2_O over N_2_ tightly linked to this^9^. Two mechanisms have been proposed for explaining the role of pH: (i) a distal impact on the genetic potential in soils through re-arrangements of the microbial community and (ii) a proximal impact driven by modulation of the direct reactions catalysing the conversion of N_2_O to N_2_ by microbial enzymes^10^. However, emissions of N_2_O are controlled at multiple levels: (i) the available genetic potential within the soil microbial community (genotype)^11^, (ii) the activation or de-activation of the potential in response to an environmental signal (transcriptional regulation controlling expression of genotype)^12^,^13^, (iii) the translation of transcripts leading to an immature or apoprotein (translational regulation)^14^, (iv) maturation of a protein resulting in an active enzyme (post-translational regulation)^14^, (v) export of enzymes when activity is not cytoplasmic (e.g. sec/tat dependent secretion as is the case for *NosZ*)^15^,^16^,^17^, and (vi) degradation or turnover rate of enzymes once active^18^. These controls cover both the production of N_2_O and the consumption, or turnover, into N_2_ by a different process. As a result emissions are limited by what may be summarized as: (i) genetic potential, (ii) transcriptional regulation, and (iii) enzymatic activity. The outcome is a complex array of regulators and processes that are likely to change across time and space.

Despite the complexity, observations support the role of both distal and proximal regulators^19^,^20^. Distal impacts by pH are proposed to be driven by selecting for community shifts at both functional and phylogenetic levels^21^ with shifts in available potential (functional gene abundances) resulting in shifts in phenotypes (observed emissions)^22^,^23^. Proximal impacts by pH provide a clearer mechanism. Low pH causes a shift in active organisms^24^, but more importantly pH disrupts the activity of the N_2_O reductase by interfering with assembly^25^,^26^,^27^. Although evidence supports the role of pH in regulating emissions and community structure^28^,^29^,^30^,^31^ studies linking all three remain sparse.

An additional consideration is the role of biodiversity in supporting ecosystem processes like N (nitrogen) cycling. It has been proposed that biodiversity is a universal regulator of ecosystem processes^32^. Although microbial studies that support the role of microbial diversity in controlling productivity^33^,^34^, N cycling^35^,^36^,^37^ and even N_2_O emissions^38^ exist, these rely on single manipulated soils or small sample sizes. However, such studies serve to establish a hypothesis that aligns with ecological theory. That is, with increasing diversity there is increased redundancy and efficiency of ecosystem processes^39^,^40^. This has been observed in other microbial studies^35^,^41^, including those associated with N_2_O emissions^42^. However, a detailed study linking gaseous emissions (NO, N_2_O and N_2_), pH and microbial diversity, over soils with varying parent materials and climates, is lacking.

In this study we aimed to link phenotypes (emission potential) to genotypes (functional potential and community composition) across 13 soils with varying pH (5.57–7.03) representing both Northern and Southern Hemisphere soils. These soils were selected as they represent the normally observed pH range in agronomic grasslands (recommended pH optima = 6.2–6.5). Using this dataset our goal was to simultaneously explore the relationship between pH, diversity and emissions. We hypothesized that the effect of pH on emissions would be linked to changes in whole communities, and not solely to denitrification functional potential. To test this, we quantified the abundance of genes involved in denitrification using quantitative PCR and metagenomic analysis, and examined their relationship with the emissions potential (N_2_O ratio = N_2_O/(NO + N_2_O + N_2_)). We also determined the microbial community composition and diversity of each soil and identified patterns linked to both changes in pH and emissions.

## Results

### pH dependent changes in emissions linked to denitrifier community size as well as to total community diversity and composition

**Figure 1:**
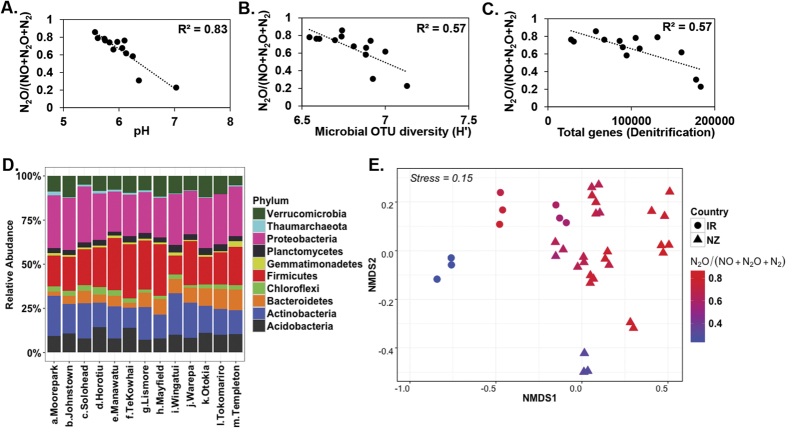
Relationship between soil pH, N_2_O emission ratio, community phylogenetic and functional potential. Relationships of N_2_O/(NO + N_2_O + N_2_) with pH (**A**), Shannon diversity based on 16S OTUs clustered at 97% sequence similarity (**B**), and total gene abundance (gene abundance per 5 ng soil DNA) for denitrification genes (*nirS, nirK, nosZI* and *nosZII*) based on qPCR (**C**). Changes in community composition at phylum level for Irish (IR) and New Zealand (NZ) soils ranked by country (a-c: IR: Ireland soils, d-m: NZ: New Zealand soils) and decreasing N_2_O emission ratio (**D**). Microbial community dissimilarities of soils with different emission profiles as determined using NMDS (Bray-Curtis) ordination (**E**).

The preferential loss of N from soils as N_2_O, or alternatively the efficiency of conversion of N_2_O to N_2_, as determined using the N_2_O ratio (N_2_O/(NO + N_2_O + N_2_)) was negatively associated with soil pH (R^2^ = 0.83, p < 0.001) (Fig. 1A). However, when individual gases produced during denitrification were considered, pH was only strongly and inversely associated with emissions of N_2_O (R^2^ = 0.62, p < 0.01), with other gases showing no clear pattern (NO [R^2^ = 0.12, p = 0.25], N_2_ [R^2^ = 0.21, p = 0.11]) (Supplementary Fig. S1). The N_2_O ratio was negatively, and pH was positively, associated with microbial diversity (R^2^ = 0.57, p < 0.01; R^2^ = 0.49, p < 0.01), as well as to total denitrification gene (*nir* & *nos*) abundance (R^2^ = 0.57, p < 0.01) (Fig. 1B,C and Supplementary Fig. S2). Across all soils the Proteobacteria, Actinobacteria and Firmicutes phyla were the dominant phyla, and represented >75% of total microbial populations in pasture soils (Fig. 1D). Comparison of samples based on 16S rRNA community composition visualised with a non-metric multidimensional scaling (NMDS) plot, using a Bray-Curtis dissimilarity matrix, also displayed a significant link to the N_2_O emission ratio and pH (Fig. 1E and Supplementary Fig. S3–S4). A Mantel test, however, supported the correlation between microbial community structure and both the N_2_O ratio (r = 0.57, p < 0.001) and pH (r = 0.61, p < 0.001). A pvclust analysis (hierarchical clustering with p-values calculated via multiscale bootstrap resampling, Supplementary Fig. S5) demonstrated that while at a 95% confidence level the clusters formed represented replicates for the same site, at lower confidence levels ( < 95%) soils could be clustered geographically (4 clusters: 1 Ireland; 3 New Zealand: Otago, Canterbury and North Island).

### pH and the N_2_O ratio correlate to distinct microbial populations

**Figure 2:**
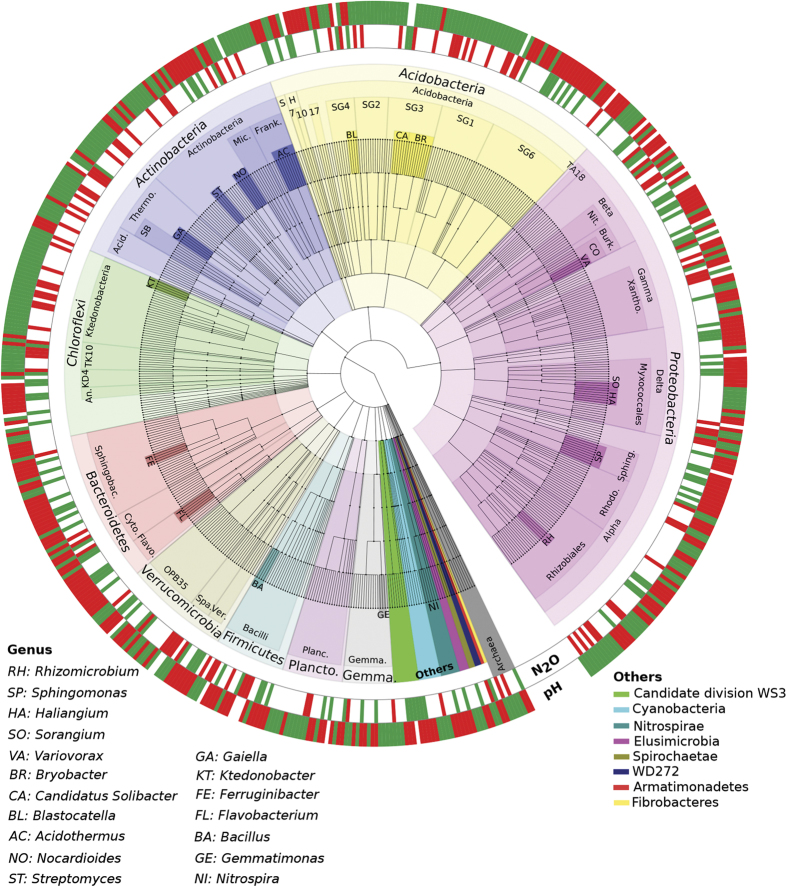
Taxonomic summary of OTUs significantly associated (p < 0.05 after BH correction; r ≥ 0.5 [Red] or ≤−0.5 [Green]) to either pH or N_2_O emissions ratio. The graph represents a cladogram of 590 OTUs. Nodes on the tree (moving outwards from centre) correspond to taxonomic level [Domain, Phylum, Class, Order, Family, Genus and OTUs]. Shaded areas of branches delineate defined taxonomic groups. Abbreviations: S, Subgroup-22; H, Holophagae; SG, 7, 10 and 17 denotes Acidobacterial orders (subgroups); Rhodo., Rhodospirillales; Sphing., Sphingomonadales; Xantho., Xanthomonadales; Burk., Burkholderiales; Nit., Nitrosomonadales; Frank., Frankiales; Mic., Micrococcales; Thermo., Thermoleophilia; Acid., Acidimicrobiia; KD4, KD4-96; An., Anaerolineae; Sphingobac., Sphingobacteriia; Cyto., Cytophagia; Flavo., Flavobacteriia; Spa., Spartobacteria; Ver., Verrucomicrobiae; Plancto., Planctomycetes; Planc., Planctomycetacia; Gemma., Gemmatimonadetes; SB, Solirubrobacterales; CO, Comamonadaceae. See Supplementary file (Table S1) for full classification.

Operational taxonomic units (OTUs at 97% sequence similarity) significantly associated to changes in emissions, or pH, were identified using Spearman’s rank correlation (Fig. 2). A total of 590 OTUs displaying both a statistically significant result (p < 0.05) and a strong effect (r ≥ 0.5 or r ≤ −0.5), based separately on either variable, were analysed. The number of detected OTUs was 2.5-fold larger for pH (554 OTUs) than for N_2_O ratio (224 OTUs) (Fig. 2). Surprisingly, the number of OTUs either positively or negatively correlated, to either variable, was relatively conserved indicating an almost 1:1 replacement of OTUs along the gradient. For pH, 49.2% of detected OTUs were positively and 50.7% were negatively correlated, whereas for the N_2_O ratio 47.8% were positively and 52.2% were negatively correlated. As a general trend, taxa showed a strongly conserved antiparallelism in relationship to pH and N_2_O ratio consistent with prior trends (Fig. 1). While certain phyla displayed conserved patterns (e.g. Chloroflexi and Bacteroidetes), all phyla had examples of contrasting responses suggesting diverse life strategies. However, certain lineages at lower taxonomic levels did present consistent patterns (e.g. class Ktedonobacteria within the Chloroflexi, Subgroup 1 & 2 of the Acidobacteria, and Frankiales within the Actinobacteria). Lineages with known functional roles associated to N cycling like the Nitrospirae (positive correlation to pH and a negative correlation to N_2_O ratio) and the Thaumarchaeota (mostly negative correlation to pH and a positive correlation to N_2_O ratio) showed clear responses. It is also worth noting that candidate phyla (WD272, WS3) as well as other poorly studied phyla (e.g. Armatimonadetes) showed strong correlations with the N_2_O ratio. For full taxonomic lineages and corresponding response to pH and emissions see Supplementary Table S1.

### Linking denitrifying genes with pH and N_2_O emissions

**Figure 3:**
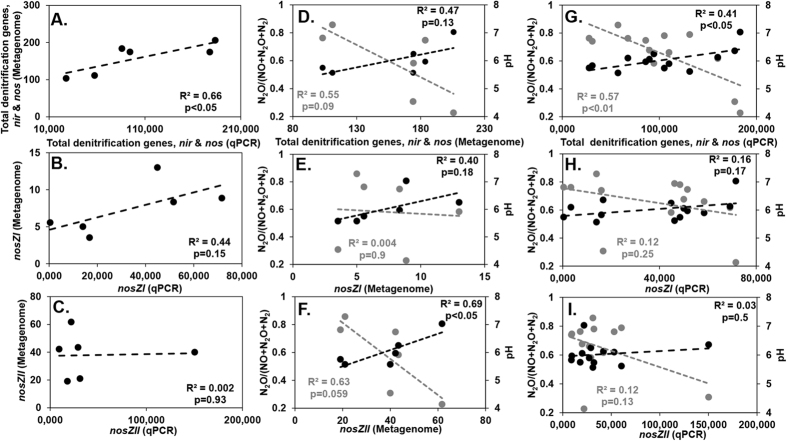
Relationship between abundance of denitrification genes (based on absolute quantification of metagenome & qPCR abundance of *nirS, nirK, nosZI, nosZII*), N_2_O/(NO + N_2_O + N_2_) and pH. (**A**–**C**) Comparison of gene abundances based on either metagenomic (i.e. gene abundance per 2.63 million reads) or qPCR analysis (gene abundance per 5 ng soil DNA) for 6 soils. (**D**–**F**) Response of total denitrification genes, *nosZ* Clade I and II abundances based on metagenomic analysis for 6 soils against N_2_O/(NO + N_2_O + N_2_) (gray) and pH (black). (**G**–**I**) Response of total denitrification genes, *nosZ* Clade I and II abundances based on qPCR analysis for all 13 soils against N_2_O/(NO + N_2_O + N_2_) (gray) and pH (black).

**Figure 4:**
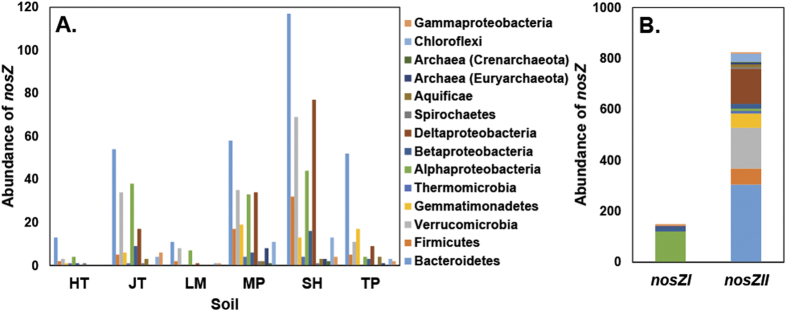
Abundance (genes per 2.63 million reads) and predicted taxonomy of nitrous oxide reductase (*nosZ*) genes by soil (3 New Zealand [HT, Horotiu; LM, Lismore; TP, Templeton] and 3 Ireland soils [JT, Johnstown; SH, Solohead; MP, Moorepark]). (**A**), and summarized by Clade (**B**), based on metagenomics analysis. Clade I: Total abundance (150), Richness (4), Shannon Diversity (0.68), Evenness (0.49). Clade II: Total abundance (824), Richness (14), Shannon Diversity (1.87), Evenness (0.46).

To determine the effect of varying pH on the genetic potential for denitrification, qPCR analysis was performed for key denitrification genes. Results confirmed a link between pH and the denitrification potential of soils (total [sum] abundance of all measured denitrification genes [*nirS, nirK, nosZI, nosZII*]). A positive association with pH (R^2^ = 0.41, p < 0.05) was observed, with an inverse response observed based on emissions (negative association with N_2_O ratio [R^2^ = 0.57, p < 0.01]) (Fig. 3). To confirm observations, and to account for potential biases associated with primers and PCR, we determined the total abundance (per 2.63 million reads per sample) of denitrification genes in metagenomes created from 6 soils (Fig. 3 and Supplementary Fig. S6). Trends based on total denitrification gene abundance were conserved between approaches (R^2^ = 0.66, p < 0.05), however, discrepancies were observed when clade specific *nosZ* gene correlations were performed. For Clade I trends were similar based on either qPCR of metagenome, although these were not statistically significant (R^2^ = 0.44). However, results for Clade II based on metagenomic data showed a strong and statistically significant link to both pH (R^2^ = 0.69, p < 0.05) and N_2_O ratio (R^2^ = 0.63, p = 0.059) that was not consistent with qPCR results. Despite low PCR efficiencies (average 66%), the abundance of *nosZ* genes belonging to Clade II were consistently higher than Clade I for both methods (~5-fold based on metagenome and 1.02-fold based on qPCR) (Figs 3, 4). Irish soils had significantly higher numbers (1.9-fold, p < 0.05, Welch’s *t*-test on metagenome data) of *nosZ* genes compared to New Zealand. It was also observed that taxonomic richness and diversity for Clade II was approximately 3-fold higher than for Clade I. A total of 11 different phyla (Bacteroidetes, Firmicutes Verrucomicrobia, Gemmatimonadetes, Thermomicrobia, Proteobacteria [Alpha, Beta, Delta and Gamma], Spirochaetes, Aquificae, Euryarchaeota, Crenarchaeota, and Chloroflexi) were identified based on *nosZ* sequences. The Bacteroidetes dominated those belonging to Clade II (*nosZ*) while the Alphaproteobacteria dominated within Clade I (Fig. 4 and Supplementary Fig. S7). We also examined the *nirS and nirK* genes individually, and found a positive association with pH (R^2^ = 0.53, p < 0.05) and negative association with N_2_O ratio (R^2^ = 0.38, p < 0.05) for *nirS* (Supplementary Fig. S8). However, no significant associations were observed for the *nirK* gene.

### Linking functional richness with pH and N_2_O emissions

To account for changes in community metabolic potential outside of those previously explored, trait (function) specific patterns, associated to pH and emissions, were explored by determining the functional richness at two different levels: general N metabolism (all N cycling related genes detected) and total functional potential (total number of different genes detected). No pattern was observed between functional richness (total functional richness as well as functional richness of N-metabolism) and pH or N_2_O emission ratio in the soil (Supplementary Fig. S9).

## Discussion

Results support the role of native soil pH in shaping community composition and diversity. Microbial community changes were associated to both geographic changes (country and region) as well as to N_2_O emissions potential, as has been described previously^21^,^43^. It is important to note that N_2_O emissions potential, or ratio, as defined in this study (N_2_O/(NO + N_2_O + N_2_)) refers to the propensity of soils to emit N_2_O over other denitrification gas intermediates. Here this is accomplished using a controlled environment where all other factors were held constant. While this does not reflect the absolute (total amount) of N lost through the process, it is possibly the best predictor of the propensity of the soils to emit N_2_O^8^,^9^. However, this potential, and the observed phenotype, can be modulated by fluctuating factors and require observations at the denitrification level through expression profiling (transcriptional/translational level) to identify real time drivers of N_2_O emissions^24^,^26^,^27^. Despite these limitations our observations highlight a conserved response to pH in both Northern and Southern Hemisphere soils. This suggests pH is part of a universally conserved mechanism selecting for both emissions and microbial communities. The range of pH observed in our soils (5.57–7.03) was sufficient to capture the range at which the N_2_O reductase and N_2_O emissions fluctuate in response to pH^26^,^44^,^45^,^46^. Soil pH controls not only the assembly of the N_2_O reductase^26^,^27^, but also alters general expression patterns^24^ and selects for shifts in microbial community composition^31^ indirectly influencing the abundance and type of functional genes in soils. Thus pH can have confounding effects due to its role in shaping the genotype, expression and eventual phenotype associated with denitrification.

While our findings support prior work, we show that of all the three measured gases only N_2_O had a significant association with pH when compared to maximum emission levels, with maximum observed N_2_O emissions decreasing with higher pH (Fig. S1). This was consistent with a lack of correlation between pH and individual denitrification genes. This is potentially due to the modular nature of denitrification^19^,^47^,^48^ where different steps within the pathway are encoded in distinct operons which do not necessarily depend on nor are associated with each other. Despite no strong correlations between emissions and denitrification specific genes, we found that of the two clades of *nosZ* gene one was dominant. Both qPCR and metagenome results show that Clade II are highly abundant, despite amplification efficiencies being poor (66%) for Clade II primers. Further, trends between metagenomic and qPCR data did not match and suggested that Clade II primers do not provide an accurate view of the abundance within our soils. Despite an apparent under representation (based on qPCR) for *nosZII*, the average Clade II/Clade I abundance ratio was >1 both for PCR-based and metagenomics analysis and is in line with prior observations of their dominance in certain soils^49^. It also aligns with reports linking the abundance of Clade II with the emissions potential of soils^50^. Our results also support the predicted diversity based on clade, with Clade II being represented in almost 3-times more phyla (Fig. 4)^51^. Despite evidence supporting the taxonomic conservation for the two clades (different *nosZ* types are found restricted to certain microbial groups)^50^,^51^,^52^ our data shows that these organisms can be associated with soils displaying contrasting pH and emissions ratios.

Despite the lack of correlation between specific denitrification genes and pH, we did observe a trend of decreasing abundance of denitrification genes and overall diversity (based on 16S analysis) with decreasing pH. The role of diversity in regulating ecosystem processes has been long debated^39^,^40^. The significance of microorganisms in this debate has only vaguely been addressed, relative to their predicted diversity^53^, despite their expected importance^54^,^55^. Available studies suggest that when specific microbial functional groups (i.e. methanotrophy vs respiration) are used to test diversity/ecosystem process relationships, significant trends can be uncovered^35^,^36^,^37^,^41^. For N_2_O, studies suggest that diversity plays a role, with decreases in diversity leading to increases in emissions^38^,^56^. Our results support and expand on those observations indicating a role for diversity-mediated responses at multiple levels (from whole community, to specific populations linked to denitrification). Though our data do not allow a mechanism to be determined, we hypothesize that an increase in diversity ensures a steady population of microbes that are capable of sustaining a process (e.g. N_2_O reduction) over a range of conditions. This diversity is still under the proximal control of regulators thus it can be modulated based on spatially and temporally controlled factors.

Identification of specific organisms responding to either pH or emissions highlighted co-varying trends. For example, while many organisms associated to changes in pH were identified as being associated to changes in emissions, not all organisms were. This implies that while certain organisms are selected by pH, they may not play a role in controlling emissions. Alternatively, some organisms that do play a role, might not be selected for by pH alone. While such correlations allow for development of new hypotheses they serve only as a first step in identifying the mechanisms controlling emissions and the role individual organisms may play. Our study also does not address the role or contributions other pathways (like nitrification) might play in regulating N_2_O emissions.

## Methods

### Sample collection and processing

Soil samples used in this study and their physio-chemical properties have been described previously^9^. Soils were selected to represent intensive agricultural grasslands with a representative pH range close to the agronomic optimum of 6.5. Briefly, soil samples were collected from 13 permanent grassland (managed agricultural) sites in Ireland (Johnstown, Moorepark, Solohead) and New Zealand (Horotiu, Lismore, Manawatu, Mayfield, Otokia, Te Kowhai, Templeton, Tokomairiro, Warepa, Wingatui), representing Northern and Southern hemisphere sites. Soil cores (n > 3) were collected randomly from each site using a corer (25 mm diameter by 100 mm long), and excluded the grass layer. For each site, replicate cores were sieved to <4 mm, composited and immediately shipped to the Norwegian University of Life Sciences, Norway for analysis. Soil samples for kinetics were stored at 4 °C in the lab until analyzed (within one week). Soils for DNA extraction were immediately frozen and stored at −20 °C until extracted. Three separate DNA extractions were performed from 0.25 g of soil material from each site (total 39) with the PowerLyzer^®^ PowerSoil^®^ DNA Isolation Kit (MoBio, Carlsbad, CA) as per manufacturer’s instructions. DNA concentration, purity and contamination with humics were assessed with a Nanodrop Spectrophotometer, ND-1000 (Thermo Scientific). DNA yields ranged between 8–21 ng/μl (median = 13; standard error = 0.6) with no detection of humic acids (median absorbance at 320 nm = 0.008; standard error = 0.0010) indicating high quality extractions.

### Gas kinetics

Gas kinetics methods were described in detail in Samad *et al*.^9^. Briefly, soils (100 g dry weight) were provided with nitrate (2 mM NH_4_NO_3_) by flooding in 500 ml filter funnels (Millipore) with 4.5 cm diameter (0.2 μm) Millipore filters at least three times for 10 minutes. To obtain a homogeneous distribution of 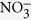 and to remove excess liquid from soils a vacuum was applied. After 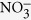 adjustment, 20 g (dry weight equivalent) of each soil was transferred to a 120 ml serum vial and sealed with an air-tight butyl-rubber septa and an aluminum crimp cap. For each site triplicate vials were prepared and incubated at 20 °C using an automated GC system^57^. The soils were first incubated for 40 h under oxic conditions and then incubated under anoxic conditions for over 200 h. The emission of NO, N_2_O and N_2_ were measured at 5 h intervals under anoxic conditions. The product ratio of N_2_O (i.e. N_2_O/(NO + N_2_O + N_2_)) was calculated and the maximum value observed during incubation for each soil was used. The maximum value represents the highest potential of each soil to emit N_2_O. While 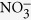 concentrations are likely to see a small increase due to nitrification of the added NH_4_^+^ during oxic incubation, resulting in soil-to-soil differences in available 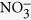 at the beginning of the anoxic incubations, these differences are unlikely to affect the kinetics of denitrification (and the product ratios) since the 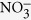 concentration applied (2 mM) was 2–3 orders of magnitude higher than *Ks* for 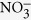 reductases^58^. Further, wetting of soils did not result in emissions with kinetics only measurable in the presence of exogenously added N.

### Quantification of bacterial community and functional gene abundance

Quantitative PCR (qPCR) was performed on all 39 extractions to determine total bacterial abundance and the abundance of four denitrification functional marker genes (*nirS, nirK, nosZ* (Clade I) & *nosZ* (Clade II)) in each soil. Reactions were performed in 96-well plates using the ViiA7 real-time PCR system (Applied Biosystems, Carlsbad, CA). Standards for qPCR were generated using a 10-fold serial dilution (10^8^ to 10^1^) of known copy numbers of pGEM-T easy (Promega, Madison, Wisconsin, USA) cloned template. All quantifications were performed using 4 technical replicates for each DNA sample loaded into the same plate, with each plate containing replicated standards and no template controls (PCR efficiencies shown in Supplementary Table S2. Amplification of *nosZ* Clade II and *nirK* targets was not possible with multiple tested polymerase brands even after optimization. As a result, two different master mixes (ABI and Thermo Scientific) were used as specific below. All reactions were performed in 20 μl volumes containing: 1× Master Mix (ABI for *nirS* & *nosZ*I or Thermo Scientific for *nirK* & *nosZII*), 0.5–1 μM of each primer (0.5 μM for *nirS* & *nosZ*I and 1 μM for *nirK* & *nosZII*), 5 ng of template DNA and autoclaved Milli-Q H_2_O to a final volume of 20 μl. Primers and qPCR conditions are summarized in Supplementary Table S2. A melt curve analysis (95 °C for 15 s, 60 °C for 1 min then increasing 0.05 °C/s (data acquisition) until 95 °C) was performed at the end of reactions to test for specificity and to confirm no amplification in the negative control. No inhibition was observed and all samples tested amplified.

### Analysis of 16S rRNA gene by amplicon sequencing

16S rRNA gene libraries were created for each DNA extraction using bacterial/archaeal primers 515F/806R targeting the V4 region of the 16S rRNA gene. Library preparation and sequencing were conducted according to the standard protocol (Version 4_13) of the Earth Microbiome Project^59^ and libraries were paired-end sequenced using the Illumina MiSeq platform. Preliminary processing was carried out in Qiime (version 1.9.0) using default parameters^60^. Sequences were clustered into Operational Taxonomic Units (OTUs) at 97% sequence similarity using the SILVA version 119 reference library^61^ and UCLUST^62^. Taxonomic classification was assigned using BLAST analysis against the SILVA database^63^. Samples were then rarified and randomly subsampled 10 times (using the Qiime command ‘multiple_rarefactions_even_depth.py’) to equal depths (16,000). Samples below that threshold were removed for a total of 38 samples retained. All 10 OTU tables per sample were subsequently merged and exported for processing in R. All downstream analysis were performed in R^64^ and described in detail in supplemental information. The 16S rRNA amplicon sequences were summited to NCBI, SRA database (SRA accession: SRP080971).

### Metagenomic sequence analysis

Six sites (Ireland: Johnstown, Moorepark, Solohead and New Zealand: Horotiu, Lismore, Templeton) representing a range of emission profiles from each country were selected for metagenomic analysis. Libraries for each metagenome were generated using the Illumina Nextera XT library preparation kit. Duplicate MiSeq 2 × 250 base paired end runs were carried out for each of the 6 samples. Sequences were submitted to and annotated using the MG-RAST server^65^. Metagenomic data is available through the MG-RAST server (ID numbers 4644147.3 to 4644142.3). Sequence counts ranged from 2,634,050- 4,851,047 before quality control. Sequences were classified taxonomically using the SILVA SSU ribosomal databases and functionally using KEGG using default settings.

### Metagenome quantification of *nosZI* and *nosZII*

To differentiate between Clade I and II variants of the *nosZ* gene, a total of 1463 sequences annotated as being *nosZ* using the KO (KEGG Orthology) database were retrieved from the metagenomic libraries in our study. In order to classify them based on clade and to provide a taxonomic placement a reference database was generated. NosZ amino acid sequences were downloaded from the FunGene database^66^ and classified as Clade I (*nosZI* [PRK02888;Tat dependent]) or Clade II (*nosZI* [nitrous_nosZ_Gp; Sec dependent]) based on conserved protein domains using CD-Search^67^. Classification was confirmed by detection of signal peptides using the PRED-TAT algorithm^68^. Taxonomy for each reference sequence was retrieved from NCBI using accession numbers associated to reference sequences. Metagenome extracted *nosZ* sequences were annotated by identifying their closest match to the reference database using BLASTX (word_size: 3, E-value:10). Matches with 60% identity and 40 amino acids coverage (cutoff) were retained and classified based on the best match. A total of 974 sequences of the original 1463 were annotated.

### Statistical analyses

All statistical analyses were performed in R^64^ using the phyloseq^69^, pvclust^70^ and vegan^71^ packages. Detailed descriptions can be found in supplemental methods.

## Additional Information

**How to cite this article**: Samad, M. S. *et al*. Phylogenetic and functional potential links pH and N_2_O emissions in pasture soils. *Sci. Rep.* **6**, 35990; doi: 10.1038/srep35990 (2016).

**Publisher’s note:** Springer Nature remains neutral with regard to jurisdictional claims in published maps and institutional affiliations.

## Supplementary Material

Supplementary Figures and legends

Supplementary Table S1
