# Erratum: Phylogenetic and functional potential links pH and N_2_O emissions in pasture soils

**DOI:** 10.1038/srep37481

**Published:** 2016-12-06

**Authors:** Md Sainur Samad, Ambarish Biswas, Lars R. Bakken, Timothy J. Clough, Cecile A. M. de Klein, Karl G. Richards, Gary J. Lanigan, Sergio E. Morales

Scientific Reports 6: Article number: 35990; 10.1038/srep35990 published online: 10 26 2016; updated: 12 06 2016.

The original version of this Article contained errors in the spelling of the author Md Sainur Samad, which was incorrectly given as M.d. Sainur Samad.

These errors have now been corrected in the PDF and HTML versions of the Article.

