# Supplementary material for: Phylogenetic and functional potential links pH and N2O emissions in pasture soils
Source: Sci Rep. 2016 Oct 26;6:35990. doi: 10.1038/srep35990 (PMC5080606; doi:10.1038/srep35990)
Supplement: Supplementary Figures and legends [file srep35990-s1.pdf]

Supplementary Information

Phylogenetic and functional potential links pH and N_2O emissions in pasture soils

Md Sainur Samad[1], Ambarish Biswas[1], Lars R. Bakken[2], Timothy J. Clough[3], Cecile A. M. de Klein[4], Karl G. Richards[5], Gary J. Lanigan[5], Sergio E. Morales[1*]

[1]Department of Microbiology and Immunology, Otago School of Medical Sciences, University of Otago, Dunedin, New Zealand
[2]Department of Environmental Sciences, Norwegian University of Life Sciences, Ås, Norway
[3]Department of Soil and Physical Sciences, Lincoln University, Lincoln, New Zealand
[4]AgResearch Invermay, Mosgiel, New Zealand
[5]Teagasc, Environmental Research Centre, Johnstown Castle, Wexford, Ireland

*Corresponding Author
E-mail: sergio.morales@otago.ac.nz (S. E. Morales)

Supplementary Figures

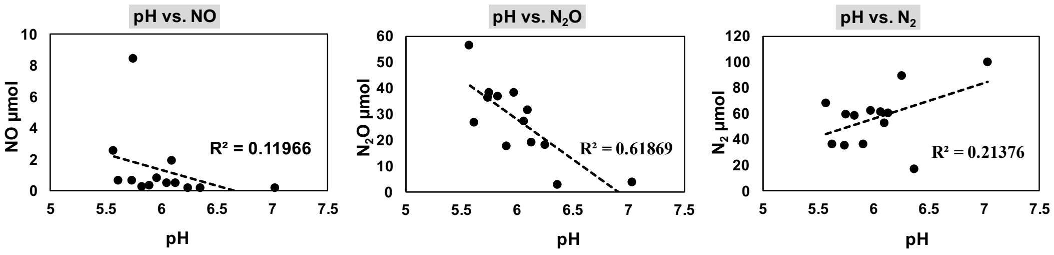

Supplementary Figure S1. Relationship between soil pH and maximum emission of NO, N_2O and N_2 under anoxic incubation for all 13 soils.

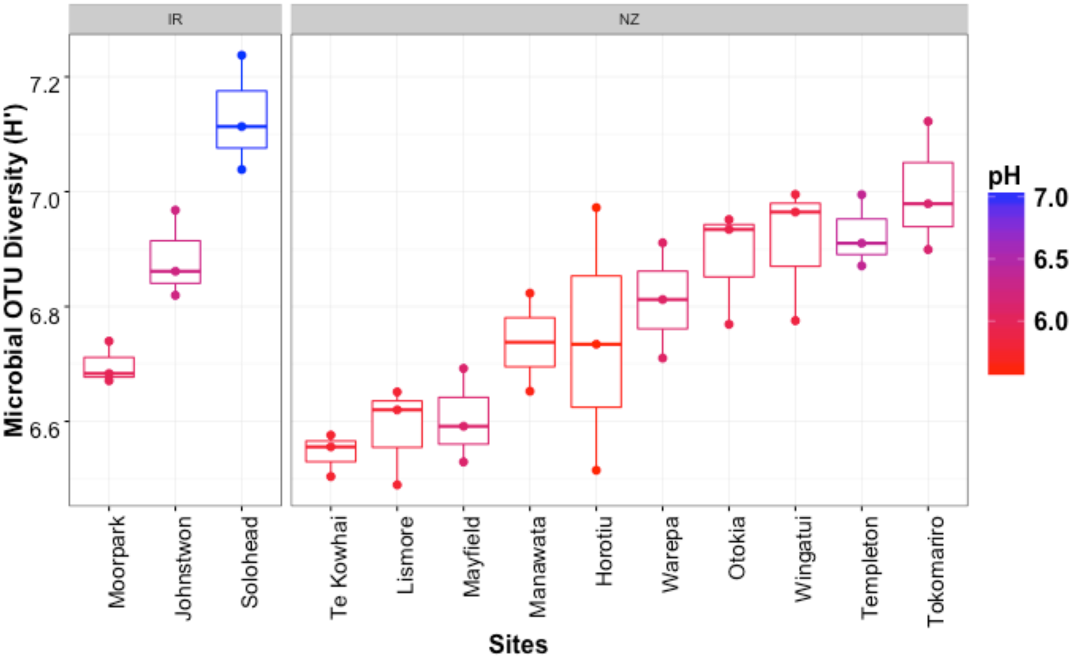

Supplementary Figure S2 Shannon diversity based on microbial OTUs across all sites for both Irish (IR) and New Zealand (NZ) soils. Color gradient denotes influence of pH ($R^2 = 0.49$, $p<0.01$).

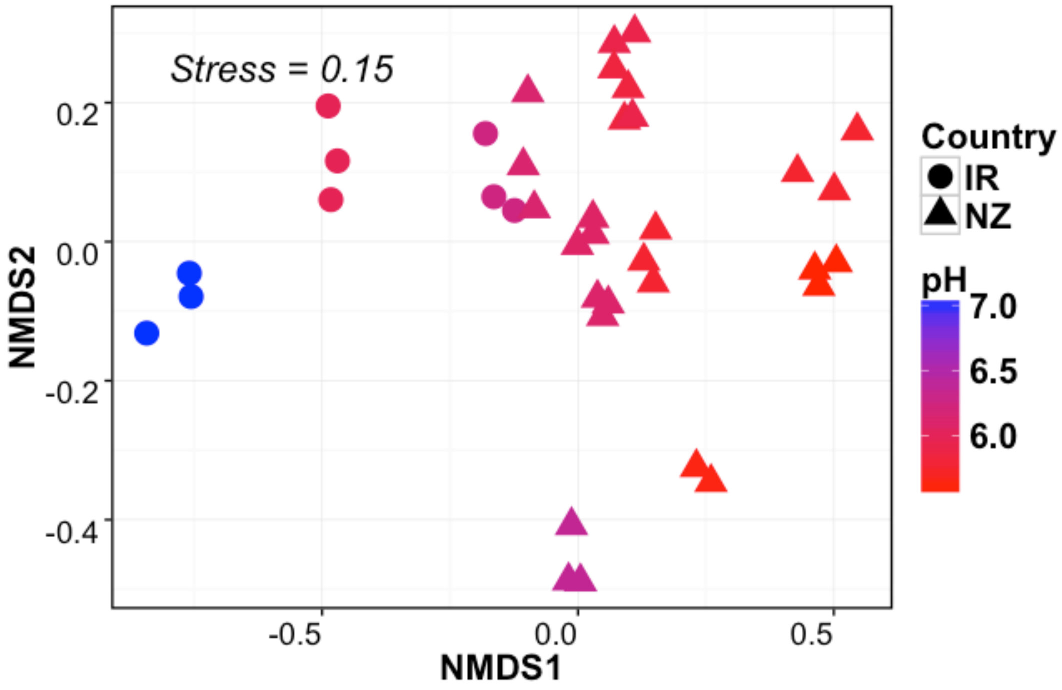

Supplementary Figure S3 Microbial community dissimilarities of soils (Irish and New Zealand) with different pH as determined using NMDS (Bray-Curtis) ordination.

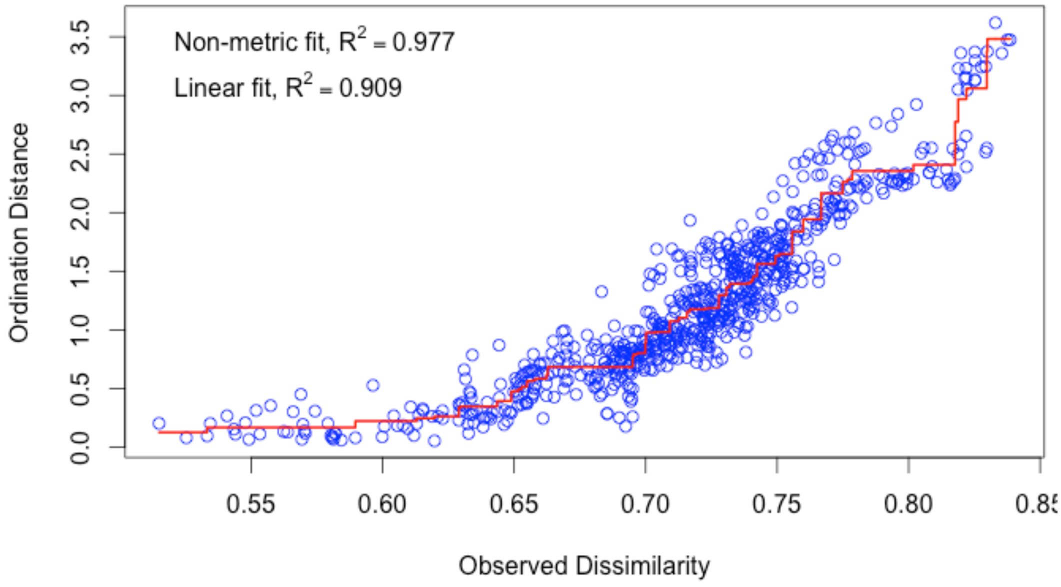

Supplementary Figure S4 Stress plot for Figure 1E

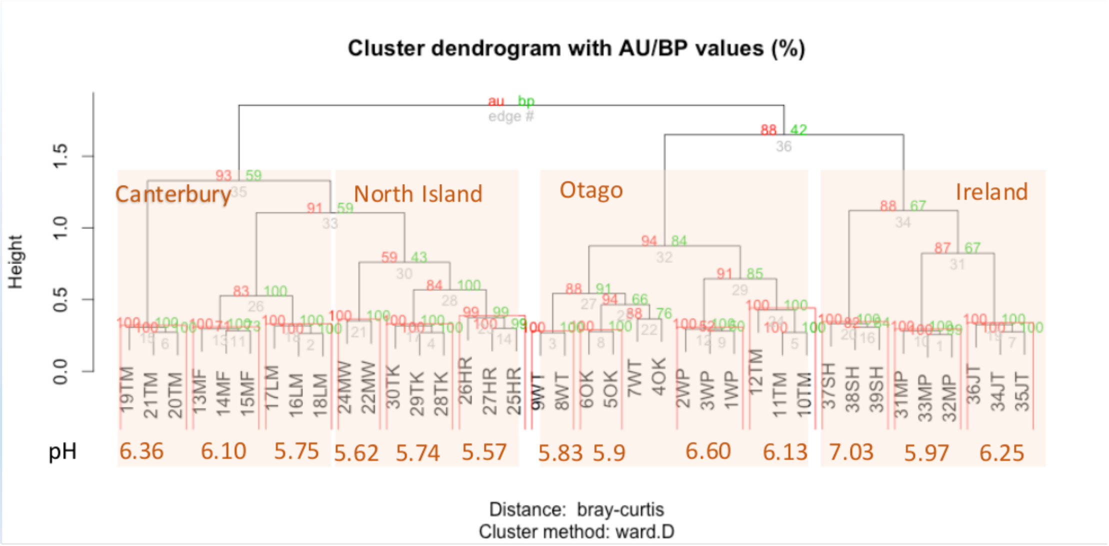

Supplementary Figure S5 Pvclust tree using Bray-Curtis distance of 16S rRNA microbial community composition and including p values for each node [AU (approximately unbiased) BP (bootstrap probability)]. Red boxes mark clusters with 95% confidence. Clusters at lower confidence are labeled by region.

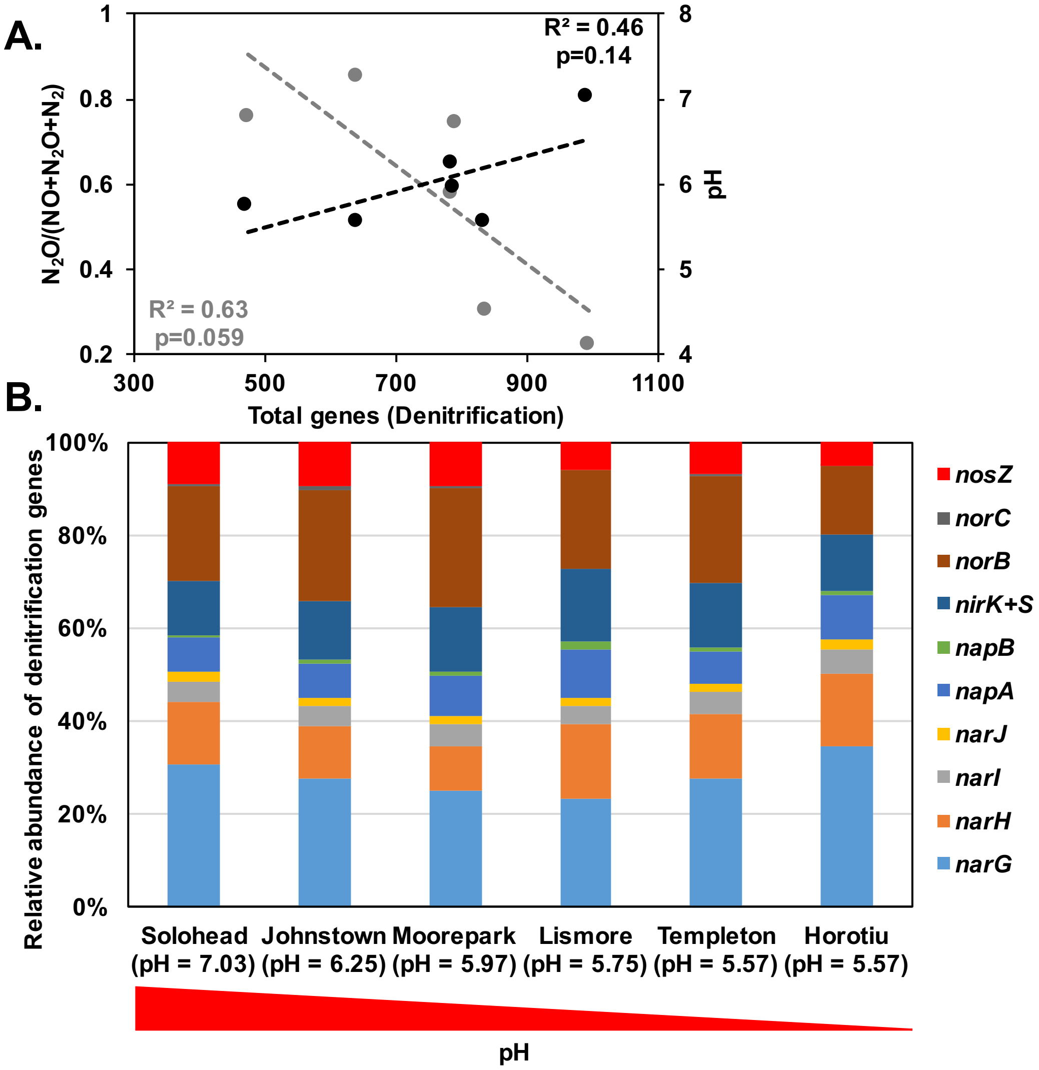

Supplementary Figure S6 Relationships between total denitrification genes (genes per 2.63 million reads) & N_2O emission ratio ($N_2O/NO+N_2O+N_2$), and total denitrification genes (genes per 2.63 million reads) & pH (A). The black circles represent the relationship between denitrification genes & pH, and the gray circles represent the relationship between denitrification genes & N_2O emissions ratio. The bottom stack bar plot shows the relative abundance of denitrification genes according to pH gradient (high to low) (B). The abundance of denitrification genes was calculated from metagenome analysis (annotation source: KO) by detection of the following genes: *nosZ, norC, norB, nirK+S, napB, napA, narJ, narI, narG.*

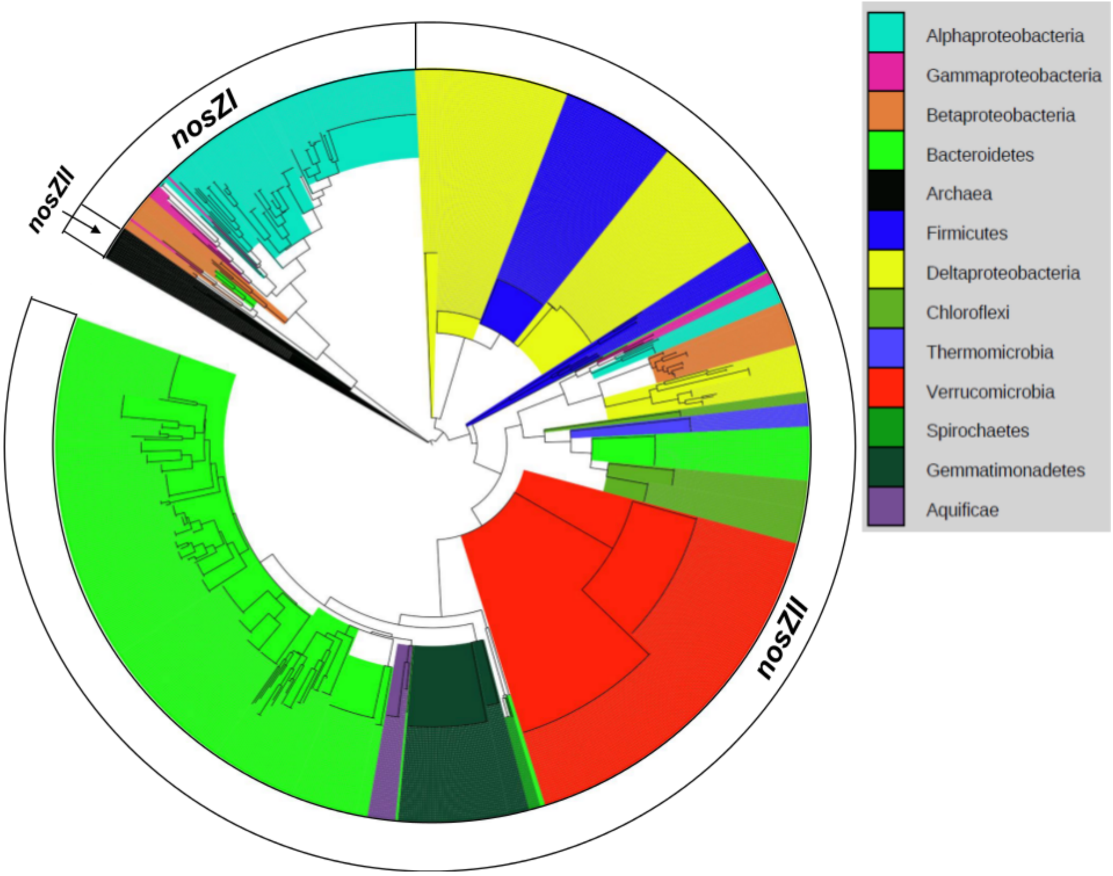

Supplementary Figure S7 Maximum likelihood phylogeny of short-length *nosZ* amino-acid sequences (129 aa) obtained from metagenomes. A multiple sequence alignment was performed with CLUSTALW on MEGA 6. After alignment, sequences were trimmed outside of conserved (90-100 %) regions (at C terminal LGPLHT--- and at N terminal ---EPH) containing approx. 129 aa sequences. The phylogenetic tree was constructed preliminary with MEGA 6 using the maximum likelihood approach and JTT matrix-based model, and finally visualized with iTOL.

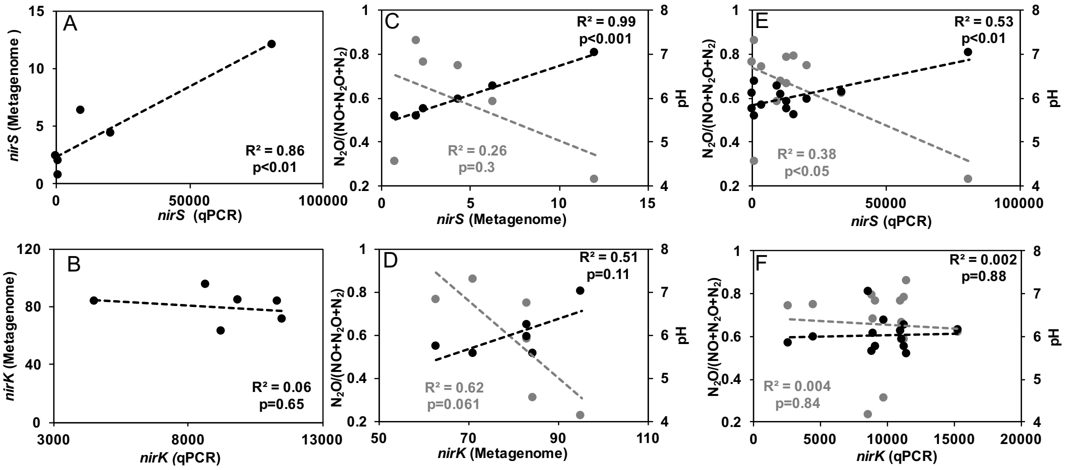

Supplementary Figure S8 Relationship between abundance of *nir* genes (based on absolute quantification of metagenome & qPCR of *nirS* & *nirK*), $N_2O/(NO+N_2O+N_2)$ and pH. (A-B) Comparison of gene abundances based on either metagenomic (i.e. gene abundance per 2.63 million reads) or qPCR analysis (gene abundance per 5 ng soil DNA) for 6 soils. (C-D) Response of *nirS* and *nirK* abundances based on metagenomic analysis for 6 soils against $N_2O/(NO+N_2O+N_2)$ (gray) and pH (black). (E-F) Response of *nirS* and *nirK* abundances based on qPCR analysis for all 13 soils against $N_2O/(NO+N_2O+N_2)$ (gray) and pH (black).

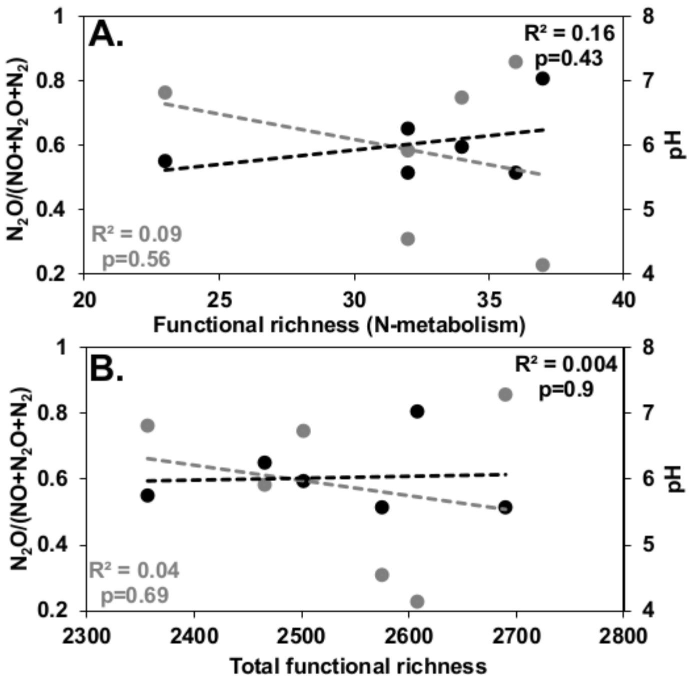

Supplementary Figure S9 Relationship between functional richness (A: at N-metabolism level and B: total functional richness), N_2O emission ratio (gray) and pH (black). The x-axis denotes richness i.e. number of different genes per 2.63 million sequence reads. The functional richness was calculated from metagenome analysis (annotation source: KO).

Supplementary Table S2 The primers used in this study

Primer	Target group	Function	Amplicon (bp)	Sequences (5´-3´)	Polymerase	Cycling conditions & data acquisition	Efficiency (%) & R^2	References
UniF<br>UniR	16S rRNA	Ribosomal RNA	180	ACTCCTACGGGAGGCAGCAGT<br>ATTACCGCGGCTGCTGGC	Fast SYBR Green Master Mix	95°C for 10 minutes, followed by 40 cycles of 10 s at 95°C, 20 s at 65°C, then followed by 20 s at 72°C for fluorescent acquisition	106 & 0.99	1
cd3AF<br>R3cd	*nirS*	NO_2^- reduction (cytochrome cd_1-containing)	425	GTSAACGTSAAGGARACSGG<br>GASTTCGGRTGSGTCTTGA	Fast SYBR Green Master Mix	95°C for 10 minutes, followed by 40 cycles of 10 s at 95°C, 20 s at 58.5°C, 20 s at 72°C then followed by 20 s at 77°C for fluorescent acquisition	94.67 & 0.99	2,3
F1aCu<br>R3Cu	*nirK*	NO_2^- reduction (Cu containing)	474	ATCATGGTSCTGCCGCG<br>GCCTCGATCAGRTTGTGGTT	Luminaris HiGreen Low ROX qPCR Master Mix	95°C for 10 minutes, followed by 40 cycles of 10 s at 95°C, 30 s at 58.5, 40 s at 72°C then followed by 20 s at 80°C for fluorescent acquisition	96.89 & 0.99	2
nosZ2F<br>nosZ2R	*nosZ* I	N_2O reduction (Tat dependent)	267	CGCRACGGCAASAAGGTSMSSGT<br>CAKRTGCAKSGCRTGGCAGAA	Fast SYBR Green Master Mix	95°C for 10 minutes, followed by 40 cycles of 10 s at 95°C, 20 s at 58.5°C, 20 s at 72°C then followed by 20 s at 75°C for fluorescent acquisition	99.3 & 0.99	4
nosZ-II-F<br>nosZ-II-R	*nosZ* II	N_2O reduction (Sec dependent)	690-720	CTIGGICCIYTKCAYAC<br>GCIGARCARAAITCBGTRC	Luminaris HiGreen Low ROX qPCR Master Mix	95°C for 10 minutes, followed by 6 cycles of 15 s at 95°C, 30 s at 60-55°C (-1°C per cycle), 30 s 72°C, and then followed by 44 cycles of 15 s at 95°C, 30 s at 54°C, 30 s at 72°C and 30 s for fluorescent data acquisition (82°C).	66.12 & 0.99	5

Supplementary Methods

Analysis of microbial community composition

The rarified OTU table (biom file) was imported into R using the Phyloseq package[6]. To account for the multiple rarifications (10 total) abundances were normalized by dividing by 10 and rounding values to whole integers using the *transform_sample_counts()* command. Taxa (OTUs) with less than 1 count were removed using the *prune_taxa()* command. Alpha diversity (Shannon and richness) were calculated using the *estimate_richness()* command.

The NMDS plot was created using a Bray-Curtis distance matrix through Phyloseq. A Mantel test was performed to test the relationship between pH, as well as N_2O emission ratio, and microbial community composition using the Vegan package[7]. To determine grouping of samples a cluster analysis was performed in using the Pvclust package (method = Ward; distance matrix = Bray-Curtis; bootstrap value, n=1000)[8]. Clusters were marked boxes (red) at 95% confidence interval.

Identifying OTUs correlated to change in pH and $N_2O/(NO+N_2O+N_2)$

In R, the Phyloseq file was transformed into a matrix. The variables (pH and $N_2O/(NO+N_2O+N_2)$) were converted into new data frames (df). A Spearmans correlation test (*cor.test(variable$pH,x, method = "spearman")*) was performed and results were further processed by adjusting p-value using a false discovery rate adjustment based on the Benjamini & Hochberg method (*p.adjust(p.vals,method = "BH")*). Results were subsetted to include only data with an adjusted p-value of < 0.05 & Rho >= 0.5 | Rho <= -0.5. OTU names were then used to subset the full Phyloseq file based on the significantly correlated OTUs using the *subset_taxa()* command. The significant OTUs and their full taxonomic classification were exported from R as a text file before visualization using Graphlan[9].

Normalization of metagenome sequences

Normalization was done based on equal number of sequence reads per sample (i.e. 2.63 million reads per sample).

Functional richness

Total functional richness (i.e. number of different functional genes) and functional richness at specific category (N-metabolism, level 3) were calculated from metagenome in MG-RAST (ID numbers 4644147.3 to 4644142.3) using KO annotation method with default MG-RAST settings (i.e. maximum e-value cutoff: 1e-5; minimum % identity cutoff: 60%; minimum alignment length cutoff: 15 aa). The functional richness data were exported from the MG-RAST and normalized based on equal number of sequence reads per sample (2.63 million reads per sample).

References

1. Hartman, A. L. *et al.* Human gut microbiome adopts an alternative state following small bowel transplantation. *Proc Nat Acad Sci* **106,** 17187–17192 (2009).
2. Throbäck, I. N., Enwall, K., Jarvis, Å. & Hallin, S. Reassessing PCR primers targeting nirS, nirK and nosZ genes for community surveys of denitrifying bacteria with DGGE. *Fems Microbiol Ecol* **49,** 401–417 (2004).
3. Yergeau, E., Kang, S., He, Z., Zhou, J. & Kowalchuk, G. A. Functional microarray analysis of nitrogen and carbon cycling genes across an Antarctic latitudinal transect. *ISME J* **1,** 163–179 (2007).
4. Henry, S., Bru, D., Stres, B., Hallet, S. & Philippot, L. Quantitative Detection of the nosZ Gene, Encoding Nitrous Oxide Reductase, and Comparison of the Abundances of 16S rRNA, *narG*, *nirK*, and *nosZ* Genes in Soils. *Appl Environ Microb* **72,** 5181–5189 (2006).
5. Jones, C. M., Graf, D. R., Bru, D., Philippot, L. & Hallin, S. The unaccounted yet abundant nitrous oxide-reducing microbial community: a potential nitrous oxide sink. **7,** 417–426 (2012).
6. McMurdie, P. J. & Holmes, S. phyloseq: an R package for reproducible interactive analysis and graphics of microbiome census data. *PLoS ONE* (2013).
7. Oksanen, J., Blanchet, F. G., Kindt, R. & Legendre, P. *Vegan: Community Ecology Package (2013) R package version 2.0-7 http://CRAN. R-project. org/package= vegan*
8. Suzuki, R. & Shimodaira, H. Pvclust: an R package for assessing the uncertainty in hierarchical clustering. *Bioinformatics* **22,** 1540–1542 (2006).
9. Asnicar, F., Weingart, G., Tickle, T. L. & Huttenhower, C. Compact graphical representation of phylogenetic data and metadata with GraPhlAn. *PeerJ* (2015). doi:10.7717/peerj.1029
